# Mechanism of Magnesium Oxide Hydration Based on the Multi-Rate Model

**DOI:** 10.3390/ma11101835

**Published:** 2018-09-27

**Authors:** Zhibo Xing, Limei Bai, Yuxin Ma, Dong Wang, Meng Li

**Affiliations:** College of Mining Engineering, North China University of Science and Technology, Tangshan 063210, China; Xing_zhibo@126.com (Z.X.); mayuxin2012vip@126.com (Y.M.); 15732526748@163.com (D.W.); lynx_li@126.com (M.L.)

**Keywords:** magnesium oxide, hydration, multi-rate model, chemical kinetics

## Abstract

The hydration of different active MgO under an unforced and ultrasonic condition was conducted in this paper to investigate the chemical kinetics model of the apparent reaction and discuss the mechanism combined with the product morphology. The dynamics fitting result shows that both the first-order and multi-rate model describe the hydration process under ultrasound well, while only the multi-rate model was right for the hydration process under an unforced condition. It indicated that the rate order of hydration was different in the hydration process under an unforced condition. The XRD and SEM show that the MgO hydration was a process of dissolution and crystallization. Part of the magnesium ions produced by dissolution of MgO did not diffuse into the solution in time, and adhered to the magnesium oxide surface and grew in situ instead. As a result, the difference in the hydration rate of the remaining MgO particles becomes wider and not in the same order (order of magnitude). The ultrasonic cavitation could prevent the in-situ growth of Mg(OH)_2_ crystal nuclei on the surface of MgO. It not only greatly improved the hydration rate of MgO and produced monodisperse Mg(OH)_2_ particles, but also made the first-order kinetics model fit the hydration process of MgO well.

## 1. Introduction

Magnesium hydroxide (Mg(OH)_2_) is a nontoxic, efficient, thermally stable, smoke suppression, and environment-friendly flame retardant [1,2,3,4,5]. It is also used to neutralize acid waste water and gases rich in sulfuric oxides, applied in the biomedical field [6,7,8,9]. This is the reason why Mg(OH)_2_ is a broad focus of the world’s attention. There are so many methods to obtain Mg(OH)_2_, such as the hydration of magnesium oxide, precipitation of a magnesium salt with an alkaline solution, the sol-gel technique, microwave-assisted technique synthesis, hydrothermal synthesis, and ammonia gas bubbling reactors, among which the magnesium oxide hydration method is the most cost-effective [10,11,12,13]. Although the process of magnesium oxide hydration seems a simple precipitation reaction, some factors need to be carefully controlled during the reaction so as to obtain magnesium hydroxide with the desired properties [14]. Otherwise, problems such as a low rate of hydration, agglomeration, and bad morphology easily occur, limiting the application prospect of magnesium hydroxide on the material properties [15,16]. Therefore, knowledge of magnesium oxide hydration kinetics is crucial for the production of high-quality magnesium hydroxide products. The hydration of magnesium oxide has been studied since the 1960s [17,18,19,20], and it has been found that the reaction step of the magnesium oxide hydration is not a simple two-step boundary expansion growth process, but a complex multi-step reaction including dissolution, diffusion, precipitation, and more. The hydration process is affected by many factors, such as the magnesium oxide properties, external force environment, hydration temperature, and nucleation site. Hydration reactions that have been determined at temperatures below 90 °C are multi-step complex reactions. In order to accurately explain the complex process of hydration, a method of establishing a growth model was adopted to simulate the hydration. Additionally, the inward diffusion and shrinking core models were used by some scholars to illustrate the hydration kinetics of crystal growth [21,22,23,24]. However, these models could not describe the process of magnesium oxide hydration well, and did not solve the problems that existed in terms of the hydration.

The reaction process and mechanism can be better understood by investigating chemical kinetics models, so as to control the reaction process through changing the experimental conditions [25,26]. From the perspective of physicochemical analysis, the hydration of magnesium oxide is a non-elementary reaction. The kinetics equation could be calculated based on the hydration rate, and the reaction process could be inferred according to the order of hydration. However, the kinetics of MgO hydration by this method have rarely been reported.

Thus, in the present paper, the kinetics model of MgO hydration with different activities under unforced and ultrasonic conditions was investigated. Combined with the characterization of the hydration products and model fitting, the process and mechanism of MgO hydration were discussed, which could solve the problem of impurity and agglomeration of the nano magnesium hydroxide products prepared by hydration and result in high-purity dispersive magnesium hydroxide.

## 2. Materials and Methods 

### 2.1. Materials

The active magnesia was calcined by high purity magnesite from Xiuyan, China [27]. The MgO content in magnesite ores was 47.11%, which was very close to the theoretical purity of 47.62%. Impurities were 0.64% CaO and 0.33% Fe_2_O_3_, thus the MgO could be used as the raw material for the hydration reaction.

Magnesite ores were crushed to a 1 mm–5 mm size for materials to calcine at high temperature until completely decomposed. Due to different calcined conditions, the activity of magnesium oxide was different [28]. Thus, magnesite was calcined in different environments (550 °C for 8 h, 600 °C for 6 h, 650 °C for 4 h, 700 °C for 3 h, 750 °C for 2.5 h, and 800 °C for 2 h, respectively) to obtain MgO of different activities, and the MgO hydration experiment was then conducted. The ethyl alcohol (analytical grade) was supplied by Tianjin Zhengda Chemicals (Tianjin, China), and the conductivity of deionized water was less than 5 μS·cm^−1^.

### 2.2. Hydration Procedure

The calcined magnesium oxide was cooled to the room temperature in a vacuum oven and ground for 3 min to fine powder, and later put into a beaker to react with deionized water in a 75 °C water bath. The hydrations were ended at 10 min, 20 min, 30 min, 40 min, 60 min, 90 min, and 120 min, respectively. The products were centrifuged, filtered, washed with absolute ethyl alcohol repeatedly, and dried at 105 °C for 5 h to completely eliminate water not chemical boned. The sample prepared was weighed to calculate the rate of MgO hydration according to Equation (1):

(1) $$\eta =\;\frac{{40(m}_{2}-m_{1})}{{18m}_{1}},$$

where *m*_1_ was the total mass before reaction/g, and *m*_2_ was the total mass after reaction/g.

The experiment included two parts: hydration under an unforced condition and hydration with ultrasonic. In order to reduce experimental error, the experiment was conducted by the parallel comparative method in all MgO hydration processes, of which the first group was the experimental group, and the other three were the repeating groups. The hydration conditions in the experimental group and repeating groups were the same. The final result was the average value of four groups.

### 2.3. Characterizations

The morphology and the phase identification of the products were characterized by scanning electron microscopy (SEM) and X-ray power diffraction (XRD), respectively. X-ray power diffraction (XRD) was conducted on an automated D/max-γ B (Japan) and monochromated CuKα radiation. The powdered samples were pressed into the holder using a glass slide. Scanning electron microscope (SEM) measurements were conducted on an automated ZEISS SUPRA 55 SAPPHIRE, Germany. The activity of the calcined products was analyzed using the chloride ion adsorption method to determine the amount of substance of chloride ion consumed (Δn_Cl_^−^), namely adsorbed by the magnesium oxide [29].

## 3. Chemical Kinetics Model

In order to investigate the hydration process of magnesium oxide and illuminate the hydration mechanism of magnesium oxide, X.J. Tang compared the hydration efficiency of the two-phase and three-phase reaction system, and discussed the hydration process in the three-phase reaction [30]. Tang found that the semi-empirical model and shrinking core model both described the hydration process of the three-phase system well, while only the semi-empirical model was right for the hydration process of the two-phase system. This showed that magnesium hydroxide only peeled off in the three-phase system, which was the important step for eliminating magnesium hydroxide agglomeration and improving the hydration rate, and these problems in the two-phase system were still not solved. According to the basic properties of the reaction and the reaction rate law, the hydration process of MgO was investigated deeply.

According to the general method, the hydration of magnesium oxide is a second-order reaction. Because of the low concentration of MgO in the reactant and the constant high concentration of H_2_O, the reaction could be regarded as a first-order reaction for the calculation. The integral form of the first-order kinetics equation is Equation (2) [31]:

(2) $$x_{A}{=x}_{A,0}\exp(-kt)$$

where *x_A_* is the concentration of reactant *A* at *t* time, *x_A_*_,0_ is the initial concentration of reactant *A*, *k* is the apparent reaction constant, and *t* is the reaction time.

The MgO hydration, which comprises a liquid reactant and solid particles to form a solid products process, is a first-order reaction system. While there is a phenomenon in the MgO hydration where some particles reacted fast, others reacted slowly. Based on first-order reaction equation, we can derive the multi-rate kinetics equation.

In the process of hydration, the activity “*n*” and the reaction rate “*v*” are important properties of the reactants. Since the properties of the reactants are distributed, this distribution can be represented by a distribution density function.

We assumed that *H*(*n*) is the distribution density function of activity. According to the definition of the density function, the ratio of the reactants whose activity is between *n* and *n* + *dn* to the whole is *H*(*n*)*dn*, and it meets the definition of the distribution density function:

(3) $$\int_{n_{min}}^{n_{max}}H\left(n\right)dn=1.$$

In Equation (3), *n_min_* is the minimum activity of the reactant, and *n_max_* is the maximum activity of the reactant.

The distribution density function of the reaction rate v of the reactant with activity n is represented by *f*(*v*|*n*). According to the definition of the distribution density function, the ratio of the reactant whose activity is *n* and reaction rate is between *v* and *v* + *dv* to reactant with activity *n* is *f*(*v*|*n*)*dv*, and it meets the definition of the distribution density function:

(4) $$\int_{v_{n,min}}^{v_{n,max}}f\left(\frac{v}{n}\right)dv=1.$$

In Equation (4), *v_n,min_* is the minimum reaction rate contents for the reactant with an activity of *n*, and *v_n,max_* is the maximum reaction rate contents for the reactant with an activity of *n*.

The *g*(*v*,*n*) represents the density distribution function of the reactants with an activity of *n* and a reaction rate of *v*, so *g*(*v*,*n*)*dvdn* denotes the fraction of the reactants in which the activity is *n* and the reaction rate is *v* in total reactants. Assuming that the total amount of the initial raw material for the hydration reaction is *W*, the content of the reactant with the property (*v*,*n*) in the hydration reaction is

(5) X(v,n)=Wg(v,n)dvdn.

In the process of the hydration reaction, the properties of the reactants are considered not to change with time, and the reactants are converted into products, which can be represented by first-order reaction kinetics. Therefore, the kinetic model for different rates of the hydration reaction can be given as:

(6) $$\frac{dX\left(v,n;t\right)}{dt}=-k\left(v,n;t\right)X\left(v,n;t\right).$$

The integral form is:

(7) X(v,n;t) = F(v,n;0)exp(−k(v,n)t),

and at time *t*, the ratio of the product to the reactant with *a*(*v*,*n*) property is

(8) $$\alpha_{v,n}\left(t\right)=\frac{F\left(v,n;0\right)-X(v,n;t)}{F(v,n;0)}.$$

Substituting Equation (7) into Equation (8), Equation (9) is obtained as follows:

(9) $$\alpha_{v,n}\left(t\right)=1-\exp(-k\left(v,n\right)t).$$

The ratio of reactants with the (*v*,*n*) property is

(10) f(v|n)dv×H(n)dn.

According to Equations (9) and (10), the conversion of reactants with the (*v*,*n*) property at time *t* can be obtained as follows:

(11) f(v|n)dv×H(n)dn×(1−exp(−k(v,n)t)).

At time *t*, the conversion rate of the reactants whose activity is from *n_min_* to *n_max_* and the reaction rate is from *v_min_* to *v_max_* is the sum of the conversion rates for each grade. It is expressed as an integral form:

(12) $$\alpha \left(t\right)=\int_{v_{min}}^{v_{max}}\int_{n_{min}}^{n_{max}}f\left(v|n\right)dv\times H\left(n\right)dn\times \left(1-\exp(-k\left(v,n\right)t)\right)\;=\int_{v_{min}}^{v_{max}}\int_{n_{min}}^{n_{max}}f\left(v|n\right)dv\times H\left(n\right)dn-\int_{v_{min}}^{v_{max}}\int_{n_{min}}^{n_{max}}f\left(v|n\right)\times H\left(n\right)\times \exp(-k\left(v,n\right)t)dvdn.$$

According to the conditional distribution density function, Equation (12) is changed to

(13) g(v,n)=f(v|n)dv×H(n)dn.

Substituting Equation (13) into Equation (12), Equation (14) is obtained as follows:

(14) $$\alpha \left(t\right)=\int_{v_{min}}^{v_{max}}\int_{n_{min}}^{n_{max}}g\left(v,n\right)dvdn-\int_{v_{min}}^{v_{max}}\int_{n_{min}}^{n_{max}}g\left(v,n\right)*\exp(-k\left(v,n\right)t)dvdn\;=1-\int_{v_{min}}^{v_{max}}\int_{n_{min}}^{n_{max}}g\left(v,n\right)*\exp(-k\left(v,n\right)t)dvdn,$$

There are often some non-reacted reactants, so the conversion rate of the reactants cannot reach 100% in the actual hydration process. Therefore, the concept of non-response is introduced into the model, and Equation (14) is revised to

(15) $$\alpha \left(t\right)=\alpha_{\infty }-\int_{v_{min}}^{v_{max}}\int_{n_{min}}^{n_{max}}\alpha_{v,n,\infty }*g\left(v,n\right)\times \exp(-k\left(v,n\right)t)dvdn,$$

where *α_∞_* is the maximum conversion of the whole reactants, and *α_v,n,__∞_* is the maximum conversion of the reactants with the (*v*,*n*) property.

### 3.1. Multi-Rate Kinetics Model

In order to facilitate the calculation, the reactants are divided into *i* grades (*i* = 1, 2, ..., *i*) according to their activity and divided into *j* grades (*j* = 1, 2, ..., *j*) according to the reaction rate. According to the central parameter fractional step method, the reaction rate constant *k* is divided into two levels, *k_s_* and *k_f_*, which respectively represent the hydration rate constant of the slow reactant and the hydration rate constant of the fast reactant. Then, Equation (14) can be decomposed into

(16) $$\alpha_{s}\left(t\right)=\alpha_{s,\infty }-\sum_{1}^{j}\sum_{1}^{i}\alpha_{v,n,s,\infty }\times f_{j}H_{i}\times {\exp(-k}_{s}t),$$

(17) $$\alpha_{f}\left(t\right){=\alpha }_{f,\infty }-\sum_{1}^{j}\sum_{1}^{i}\alpha_{v,n,f,\infty }\times f_{j}H_{i}\times {\exp(-k}_{f}t),$$

where *α_s_*(*t*) is the conversion rate of slow reactants at time *t*, *α_s,__∞_* is the maximum conversion rate of slow reactants, *α_f_*(*t*) is the extent of fast conversion, and *α_f,__∞_* is the maximum conversion rate of fast reactants.

(18) $$\alpha_{s,\infty }=\overset{j}{\underset{1}{\sum }}\overset{i}{\underset{1}{\sum }}f_{j}H_{i}\times \alpha_{v,n,s,\infty }$$

(19) $$\alpha_{f,\infty }=\overset{j}{\underset{1}{\sum }}\overset{i}{\underset{1}{\sum }}f_{j}H_{i}\times \alpha_{v,n,f,\infty }$$

Substituting Equation (18) into Equation (17) and Equation (19) into Equation (17), the Equations (20) and (21) are obtained as follows:

(20) $$\alpha_{s}\left(t\right)=\alpha_{s,\infty }\left({1-\exp(-k}_{s}t)\right)$$

(21) $$\alpha_{f}\left(t\right)=\alpha_{f,\infty }\left({1-\exp(-k}_{f}t)\right).$$

The multi-rate kinetics model of magnesium oxide hydration is updated by

(22) $$\alpha \left(t\right)=\left(\alpha_{s,\infty }{+\alpha }_{f,\infty }\right)-\left(\alpha_{s,\infty }{\exp(-k}_{s}t){+\alpha }_{f,\infty }{\exp(-k}_{f}t)\right)\;=\alpha_{\infty }-\left[\alpha_{f,\infty }{\exp(-k}_{f}t){+(\alpha }_{\infty }-\alpha_{f,\infty }{)\;\exp(-k}_{f}t)\right],$$

where *k_f_* is the apparent fast reaction constant, and *k_s_* is the apparent slow reaction constant.

### 3.2. First-Order Kinetics Model

When the hydration rates of magnesium oxide in the system were the same, the first-order kinetics equation was adjusted to Equation (23):

(23) $$\alpha \left(t\right)=\alpha_{\infty }\left(1-\exp(-kt)\right).$$

In Equation (23), α*_∞_* is the maximum conversion of MgO, and *k* is the apparent reaction constant.

## 4. Results and Discussion

### 4.1. The Model of Hydration under Unforced Condition

**Figure 1:**
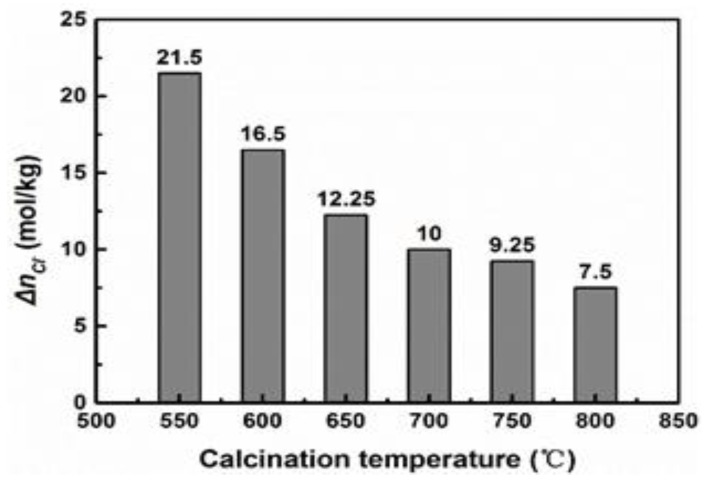
Experimental result Δn_Cl_^−^ of different active magnesium oxide.

Based on a systematic study of the calcination condition in the early stage [28,29], it was found that with an increase in calcination temperature, the decomposition speed of magnesite increased, but the maximum activity of magnesium oxide decreased. And at constant calcination temperature, the activity of magnesium oxide increased firstly with an increasing residence time up to a certain point, and then decreased. The activity of calcined products in this paper is shown in Figure 1. The data shows that the activity of MgO decreased with the increase of calcination temperature. The magnesium oxide obtained at 550 °C was the most active among all products according to the chloride ion adsorption method, and the MgO obtained at 800 °C had the lowest activity.

**Figure 2:**
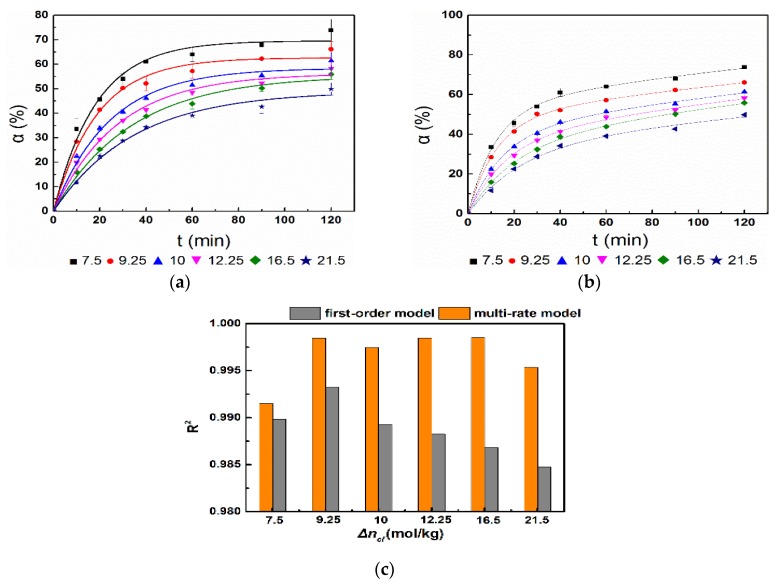
Simulation of the magnesium oxide hydration rate under an unforced condition: (**a**) First-order model; (**b**) multi-rate model; (**c**) correlation coefficient.

The simulation results of different active magnesium oxide hydration rates are shown in Figure 2. No matter what the activity of magnesium oxide was, the hydration process underwent a rapid growth followed by a slowing down, as depicted in Figure 2a,b and the curves’ inflection points appeared at the hydration time of 60 min.

Figure 2c illustrates that the R^2^ of the first-order kinetic model of magnesium oxide hydration was below 0.99 when the activity of magnesium oxide was higher than 10, and when the activity was less than 10, the R^2^ could reach 0.99 or higher. When the activity of magnesium oxide was tested, the R^2^ of the magnesium oxide hydration kinetic model was above 0.99. Therefore, the process of magnesium oxide hydration was consistent with the multi-rate kinetics model when the activity of magnesium oxide was high. The hydration process of magnesium oxide was not only consistent with the multi-rate kinetics model, but also consistent with the first-order kinetics model, when the activity of magnesium oxide was low.

**Figure 3:**
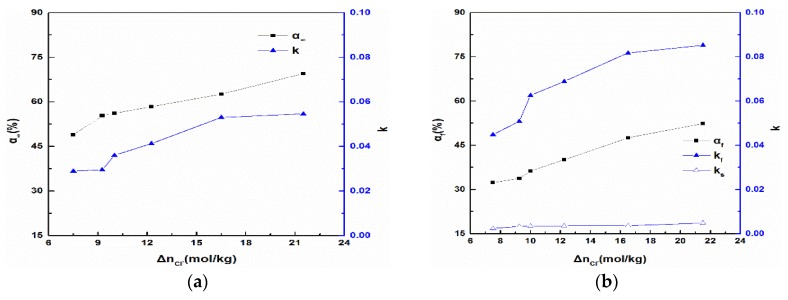
Dynamics fitted results of hydration process data: (**a**) first-order model; (**b**) multi-rate model.

Additionally, the associated parameter values of the first-order model and multi-rate model are shown as Figure 3. It could be found that the *α_∞_* of magnesium oxide plummeted and the k decreased to varying degrees with the decrease of the magnesium oxide activity, as shown in Figure 3a. When the MgO activity Δn_Cl_^−^ decreased from 21.50 mol/kg to 16.50 mol/kg, the α*_∞_* dropped from 69.50% to 62.60% and the k fell from 0.05458 to 0.05303. It could be seen that the activity Δn_Cl_^−^ and *α_∞_* respectively decreased by 23.26% and 9.21%; however, the k only decreased by 0.41%, which was a very small decline. While the magnesium oxide activity Δn_Cl_^−^ decreased from 16.5 mol/kg to 7.5 mol/kg, the *k* fell from 0.05303 to 0.02890, and the Δn_Cl_^−^ and k were reduced by 54.55% and 45.50%, respectively. In the Figure 3b, the drop of the fast reaction constant *k_f_* was quicker than the slow reaction constant *k_s_*. Besides, the change of constant was positively correlated with the activity of magnesia, whether it was a fast reaction or slow reaction.

When the activity of MgO was as high as 21.5 mol/kg, the first-order kinetics model was found to be inadequate to describe the hydration of MgO with high standard error, and the *k* value was close to that of MgO activity at 16 mol/kg. With the reduction of magnesium oxide activity, the first-order kinetics model gradually described magnesium oxide hydration better. 

**Figure 4:**
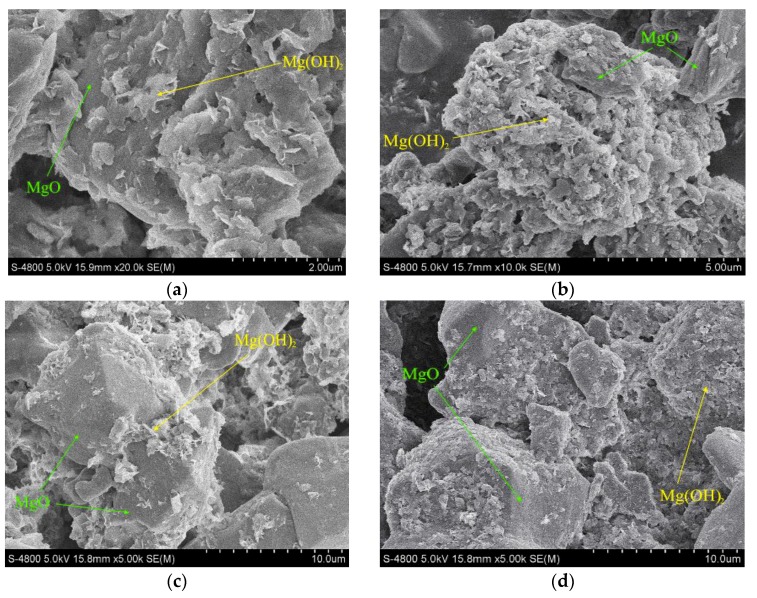
SEM images of hydration products: (**a**) Δn_Cl_^−^ = 21.5 mol/kg, t = 10 min; (**b**) Δn_Cl_^−^ = 21.5 mol/kg, t = 40 min; (**c**) Δn_Cl_^−^ = 7.5 mol/kg, t = 10 min; (**d**) Δn_Cl_^−^ = 7.5 mol/kg, t = 40 min.

Because of the different activity magnesium oxide used in the present study, the hydroxide would be formed in different times (Figure 4). Some magnesium hydroxide had been on the surface of magnesium oxide, and finally covered the whole magnesium oxide, which greatly reduced the purity of the final product, magnesium hydroxide. The growth direction of Mg(OH)_2_ was hampered by the attachment surface, and the morphology of the crystal became irregular. For the magnesium oxide with an activity of 21.5 mol/kg, the bulk of active magnesium oxide and plate-like Mg(OH)_2_ were observed in Figure 4a at 10 min, and the conversion rate of MgO was 33.54%. With the extension of reaction time, the hydration rate of magnesium oxide and the content of sheet magnesium hydroxide increased continuously. After 40 min of hydration (Figure 4b), the surface of magnesium oxide particles was tightly wrapped by Mg(OH)_2_, resulting in a smaller area of magnesium oxide in contact with the solution. At this time, the conversion rate of magnesium oxide was 61.06%. The hydrated time was prolonged, and the conversion rate of magnesium oxide was slowed down since the package of the magnesium hydroxide particles prevented the internal magnesium oxide from hydrating continuously. 

Compared to the product of magnesium oxide with an activity of 7.5 mol/kg (Figure 4c), the content of magnesium hydroxide flake particles in the product of magnesium oxide hydration with an activity of 21.5 mol/kg was higher at 10 min. When the hydration time was extended to 40 min, the content of flake magnesium hydroxide particles in the two active magnesium oxide hydration products increased significantly. Compared to the product of magnesium oxide with an activity of 7.5 mol/kg, not only was the hydration rate of magnesium oxide with an activity of 21.5 mol/kg higher, but also part of the magnesium hydroxide was relatively densely packed on the surface of magnesium oxide particles. The hydration rate of high active magnesium oxide was more affected by the wraps of magnesium hydroxide particles. Therefore, the higher the magnesium oxide activity, the greater the effect of magnesium hydroxide in-situ on the hydration rate of magnesium oxide, and the hydration was incompatible with the first-order kinetics model etc.

**Figure 5:**
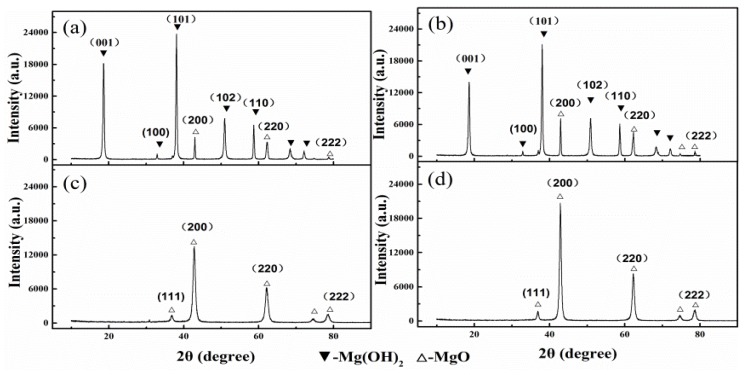
XRD patterns of calcined products: (**a**) Δn_Cl_^−^ = 21.5 mol/kg, t = 120 min; (**b**) Δn_Cl_^−^ = 7.5 mol/kg, t = 120 min; (**c**) Δn_Cl_^−^ = 21.5 mol/kg, t = 0 min; (**d**) Δn_Cl_^−^ = 7.5 mol/kg, t = 0 min.

The XRD results (Figure 5) revealed that none of the different active MgO was converted to Mg(OH)_2_ completely, which matched well with the SEM analysis results. The presence of the (200) face and (220) face of magnesium oxide could be clearly observed in Figure 5a,b which illustrated that the two active magnesium oxide was not completely converted into magnesium hydroxide during 120 min. Furthermore, the intensity of magnesium oxide peaks in Figure 5b was higher than that of Figure 5a, indicating that the content of magnesium oxide (Figure 5b) was relatively greater. At the same time, the (200) face intensity of magnesium oxide (Figure 5d) with an activity of 7.5 mol/kg was significantly higher than that of magnesium oxide (Figure 5c) with an activity of 21.5 mol/kg. The full width at half maximum of the (200) face of the former (Figure 5d) was narrower than that of the latter (Figure 5c). It further proved that the active magnesium oxide had many lattice defects and was prone to hydrate.

### 4.2. The Model of Hydration under Ultrasound Condition

**Figure 6:**
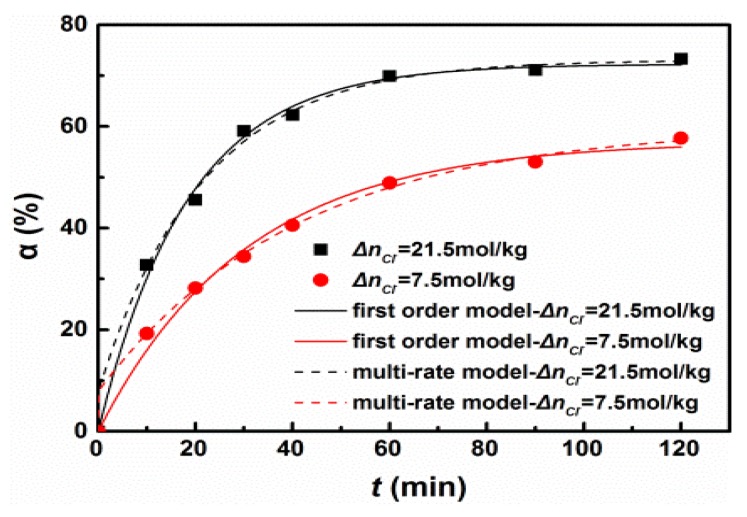
Degree of magnesium oxide hydration under an ultrasound condition.

**Figure 7:**
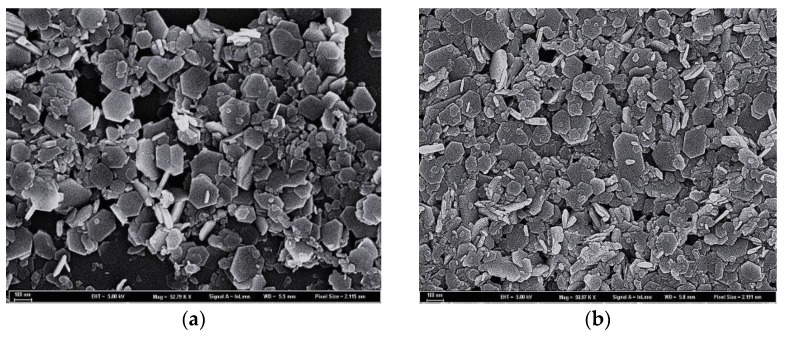
SEM images of the products of hydration in the ultrasound field at 120 min: (**a**) Δn_Cl_^−^ = 21.5 mol/kg; (**b**) Δn_Cl_^−^ = 7.5 mol/kg.

In order to eliminate the phenomenon of package and agglomeration in the hydration process, the experiment was conducted under ultrasonic irradiation. The magnesium oxide with activity of 21.5 mol/kg and 7.5 mol/kg hydrated under an ultrasonic condition. The kinetics fitting results of the hydration rate are shown in Figure 6 and the morphology of hydration products is shown in Figure 7. Both the first-order kinetics model and multi-rate kinetics model well-described the data of magnesium oxide hydration under the ultrasound condition, and the R^2^ was greater than 0.99. The hydration under an ultrasound condition belonged to the first-order kinetics model. Although the degree of the hydration of the rapid reaction period under an unforced and ultrasonic condition was similar, the degree of hydration under an ultrasonic condition was slightly higher than that under an unforced condition in general. It implied that the energy of ultrasound eliminated the phenomenon of multi-rate and improved the degree of hydration.

The SEM images of the integrated ultrasonic hydration product (Figure 7) show that the morphology of hydration products Δn_Cl_^−^ = 21.5 mol/kg and Δn_Cl_^−^ = 7.5 mol/kg) was not of a uniform-sized plate, in which parts were hexagonal platelet-shaped. Neither of them packaged and obtained dispersive flake Mg(OH)_2_. It illustrated that ultrasound could prevent magnesium hydroxide from enveloping magnesium oxide, promote the production of magnesium hydroxide particles, reduce the difference of hydration rate, and also make the reactants have the same order. 

Under an ultrasonic reaction condition, the hydroxide particle covered on the MgO surface was exfoliated to the solution by the ultrasonic cavitation. Magnesium oxide, which was exposed to the solution, continued to dissolve, promoting the formation of the growth unit. The ultrasonic cavitation could promote the diffusion of Mg^2+^ in solution, so it could carry out the generation of new nucleation in solution and give the space to leave all crystal faces and vertices of the growth unit free.

### 4.3. Mechanism of MgO Hydration

Magnesium oxide hydration can be described by the following principal dissolution steps and precipitation step at temperatures lower than 90 °C [14,24,32,33]:

MgO(s) + H_2_O(l)→MgOH^+^(surface) + OH^−^(aq), (24)

MgOH^+^(surface) + OH^−^(aq)→MgOH^+^•OH^−^(surface), (25)

MgOH^+^•OH^−^(surface)→Mg^2+^(aq) + 2OH^−^(aq), (26)

Mg^2+^(aq) + 6OH^−^(aq)→Mg(OH)_6_^4−^(aq), (27)

Mg(OH)_6_^4^^−^(aq) + Mg^2+^(aq)→(Mg(OH)_2_)_2_(OH)_2_^2−^(aq), (28)

(Mg(OH)_2_)_2_(OH)_2_^2^^−^(aq) + Mg^2+^(aq)→(Mg(OH)_2_)_3_(s), (29)

(Mg(OH)_2_)_3n−1_(OH)_2_^2−^(aq) + Mg^2+^(aq)→(Mg(OH)_2_)_3n_(s). (30)

In the hydration process, Mg^2+^ required for Mg(OH)_2_ precipitation is released from the dissolution of MgO. When the concentration of Mg^2+^ and OH^−^ in the solution reaches a certain degree of saturation, they begin to nucleate, resulting in close coupling between the precipitation and dissolution reactions. 

The precipitation reaction of magnesium hydroxide consumes the product Mg^2+^ of the dissolution reaction, which facilitates the dissolution. The adverse effect is that generated Mg(OH)_2_ covers the surface of MgO, thus isolating the reactive surface from the reactive components and restraining the hydration. Therefore, the controlling step in the whole hydration process is MgO diffusion.

**Figure 8:**
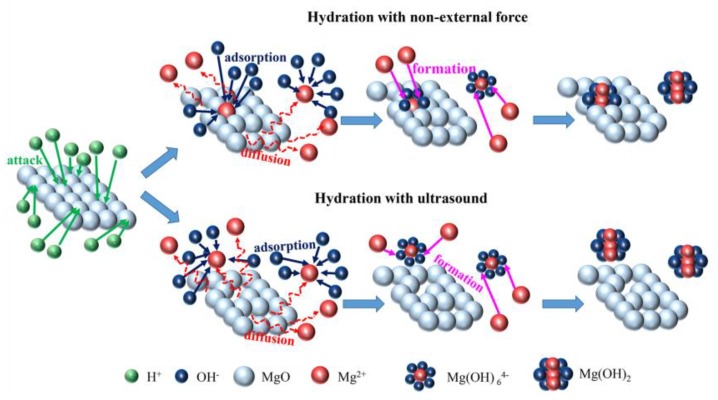
The mechanism of MgO hydration.

According to the growth unit model of the anion coordination polyhedron and the dissolution and precipitation reaction (Figure 8), water is reduced to OH^−^ and H^+^ by electrolysis, and then H^+^ acts on the surface of MgO to generate the Mg^2+^ ion. Following this, the Mg^2+^ ion absorbs the nearly ionized OH^−^, and Mg(OH)_6_^4−^ growth units are formed. As the reaction proceeds, growth units combine to produce magnesium hydroxide. During the whole process, the necessary ions of precipitation are Mg^2+^ and OH^−^ released during the reaction.

During the hydration under an unforced condition, Mg^2+^ dissolved on the MgO surface was in the solution or attached to the surface. Mg(OH)_6_^4−^ growth units formed by the attached Mg^2+^ and OH^−^ began to grow in-situ. Since magnesium hydroxide grew and crystallized on the surface of part of magnesium oxide, it enveloped the internal magnesium oxide and hindered the contact with the components in the solution, meaning that the magnesium oxide could not continue to undergo a dissolution reaction, thus inhibiting the hydration reaction. Combined with the hydration kinetics model, it could be considered that this is the reason why the hydration rate of active magnesium oxide varied under an unforced condition.

During the hydration under an ultrasonic condition, ultrasonic cavitation improved the dissolution of MgO and the diffusion of Mg^2+^ in the solution, prevented the growth of Mg(OH)_6_^4−^ growth units on the surface of MgO in-situ, provided energy for the magnesium hydroxide precipitation reaction, and accelerated the reaction. Furthermore, the ultrasonic cavitation broke the gravity between the MgO and Mg(OH)_2_, Mg(OH)_2_ was peeled off, and the fresh MgO was exposed in the solution to dissolve. Therefore, different parts of magnesium oxide have the same dissolution probability or have the same order of hydration rate under an ultrasonic condition. Magnesium oxide gradually dissolves from the outside to the inside, releasing Mg^2+^, so that the Mg(OH)_6_^4−^ growth units grow in order along the surface of magnesium hydroxide, and grow into dispersive platelets of magnesium hydroxide, effectively preventing the reunion of magnesium oxide. As for the irregular morphology of magnesium hydroxide obtained in the ultrasound field, the method for entirely forming hexagonal platelets needs to be investigated further.

## 5. Conclusions

In summary, the kinetics and mechanism of hydration under unforced and ultrasonic conditions were investigated. The results indicated that the in-situ growth hydroxide was the main reason for the low rate of magnesium oxide and the serious agglomeration of hydration products. Ultrasonic cavitation eliminated the in-situ growth of magnesium hydroxide, so that the hydration kinetics model was transformed from a multi-rate model with different rate orders under an unforced condition to a first-order model with the same rate order under an ultrasonic condition. The mechanism of MgO hydration could conclude as follows: (1) MgO dissolution; (2) Mg^2+^ diffusion; (3) Mg(OH)_6_^4^^−^ growth unit formation and overlay; (4) Mg(OH)_2_ precipitation. It can be concluded that step (2) controls the rate of hydration and the morphology of the product.
